# Effects of Road Interactions on Mate‐Searching Movements and Mate Location Success in an Imperiled Pit Viper (*Crotalus horridus*)

**DOI:** 10.1002/ece3.71102

**Published:** 2025-04-07

**Authors:** Elizabeth J. Noble, Anna F. Tipton, Morgan L. Thompson, John R. Powers, Amber A. Stubbs, William L. Tillett, Jorge A. Vázquez Diosdado, Dominic L. DeSantis

**Affiliations:** ^1^ Department of Biological & Environmental Sciences Georgia College & State University Milledgeville Georgia USA; ^2^ Department of Biological Sciences University of Texas at El Paso El Paso Texas USA; ^3^ Department of Biological Sciences Northern Arizona University Flagstaff Arizona USA; ^4^ School of Veterinary Medicine and Science University of Nottingham Leicestershire UK

**Keywords:** accelerometry, behavior, movement ecology, radio telemetry, road ecology, snake

## Abstract

For many species, male mate‐searching movements are among the primary determinants of mate location success, and males often incur significant risks associated with elevated movement during reproductive seasons. In an increasingly human‐modified world, this often includes more frequent interactions with anthropogenic landscape features, such as roadways. While road mortality represents the most direct and easily measured cost of road interactions, pervasive indirect or sub‐lethal costs could carry significant fitness consequences that are more difficult to quantify. We leveraged radio telemetry and accelerometry monitoring to explore the associations between seasonal movement strategies, mate location success, and road interactions in Timber Rattlesnakes ( *Crotalus horridus* ) from the piedmont ecoregion of Georgia, USA, where populations are in decline. Males, but not females, significantly elevated measures of movement and the frequency of road interactions during mating seasons, supporting our predictions. By using accelerometers to evaluate fine‐scale activity responses to roads, we identified a positive association between road interactions and male activity that was conserved across behavioral seasons. Unexpectedly, there were no associations detected between mate location success and road interactions. However, underlying variation in male movement measures revealed differing associations between movement and mate location success within road interaction categories. We discuss the possible roles of chemosensory disruption and road avoidance in this system, while emphasizing the need for further sampling and targeted field experiments to boost observations of road interactions and refine our understanding of these associations. Ultimately, our results are the first to directly quantify the relationship between elevated male movement in mate searching efforts and increased road interactions by longitudinally monitored rattlesnakes, despite this trend being widely recognized through observational studies of road mortality. For *C. horridus* , specifically, roads are implicated as a leading driver of declines range‐wide, and our results further highlight mating seasons as a window of highest vulnerability.

## Graphical abstract

Our study aimed to provide additional evidence for sub‐lethal effects of roadways on animal behavior. In this case, we found strong support for increased road interactions by males during mating seasons and the possibility of reduced mate searching efficiency by those males interacting with roads that warrants future investigation.

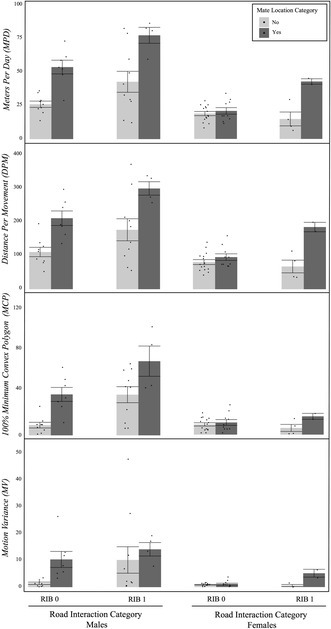

## 1 Introduction

Movement is fundamental to the biology and ecology of animals, as individuals need to move across space and time in efforts to acquire critical, fitness‐determining resources, such as food, water, shelter, and mating partners. A complex suite of intrinsic and extrinsic factors can interact to produce variation in movement strategies within and among individuals through shifting motivational states and differing cost‐benefit trade‐offs (Shepard 2008; Nathan et al. 2008). One well‐established example of intrinsic factors producing variation in animal movement behavior is seen in vertebrate mating systems based on male searching behavior. In such examples, males are under strong positive selection to search widely for reproductive females, thereby trading off elevated movement costs for greater reproductive success (Emlen and Oring 1977; Parker 1984; Fromhage et al. 2016; DeSantis et al. 2019). However, these season‐ and sex‐specific strategies have evolved outside the context of increasingly prominent human modifications to natural landscapes. Understanding how individuals and populations respond to these novel extrinsic factors is key to interpreting resilience in the face of change and could be critical for effective conservation or management actions (Parsons 2016; Fraser et al. 2018).

Of the many pervasive human influences on landscape composition, roadways are among the most widespread and disruptive (Oxley et al. 1974; Forman and Alexander 1998; Jaeger et al. 2005; Reynolds‐Hogland et al. 2005; Fahrig and Rytwinski 2009). Roads negatively impact populations most directly through vehicle‐induced mortality or intentional killing on or near roadways (Ashley and Robinson 1996; Shepard, Dreslik, et al. 2008; Quintero‐Ángel et al. 2012; Rytwinski and Fahrig 2012; Jenkins et al. 2021). However, not all roadways feature high traffic volumes and associated high direct mortality rates, and there is a growing body of evidence indicating that non‐lethal or indirect effects of roads on animal behavior can also carry significant fitness consequences (Lodé 2000; Forman et al. 2003; Keller and Largiader 2003; Shine et al. 2004; Clark et al. 2010; Holderegger and Di Giulio 2010; Robson and Blouin‐Demers 2013; Ware et al. 2015; Mata et al. 2017; Lomas et al. 2019). These indirect impacts often manifest as disruptions to animal movement behavior, as roadways fragment and degrade habitats (Forman et al. 2003; Mata et al. 2017), acting as semi‐permeable barriers to movement (Lomas et al. 2019) that can impede gene flow and reduce population viability (Lodé 2000; Keller and Largiader 2003; Shine et al. 2004; Holderegger and Di Giulio 2010; Clark et al. 2010; Robson and Blouin‐Demers 2013; Ware et al. 2015). Moreover, increased human presence, vehicular traffic, and noise associated with roads have been linked to reduced fitness and road avoidance (Ware et al. 2015; Ng et al. 2019; Zhou et al. 2020). There is also evidence from a wide diversity of vertebrate taxa for the effects of roads on movement behavior varying relative to key intrinsic factors, such as sex and seasonal motivational states (Aresco 2005; Montgomery et al. 2013; Baek et al. 2023). Additionally, the functional response to roads is undoubtedly landscape‐dependent, with road densities and adjacent habitat characteristics mediating how animals move in relation to roadways (Beyer et al. 2013). A detailed understanding of how roads impact populations, therefore, requires careful consideration of sex and seasonal motivational states when evaluating the relationship between road interactions and movement behavior.

Large‐bodied snakes, such as most pit vipers (Viperidae; Crotalinae), represent uniquely tractable models for interpreting the interacting effects of sex, season, and roadways on movement behavior (Row et al. 2012). In the male search‐based mating system characteristic of most pit vipers, males typically increase movement and space use during the mating season (i.e., mate‐searching movements) relative to the non‐mating season (Duvall et al. 1993; Madsen et al. 1993; DeSantis et al. 2019). These movement patterns are considered to be one of the primary determinants of male reproductive success, and therefore, male movement during mating periods is under strong positive selection (Duvall et al. 1993; Madsen et al. 1993; Clark et al. 2014; DeSantis et al. 2019). However, increased movement also comes with increased risks, including elevated energy expenditure, more frequent encounters with predators (Clark et al. 2010; Lomas et al. 2019), and, in an increasingly human‐modified world, the potential for increased interactions with anthropogenic features, such as roadways (Jones et al. 2022). Direct and indirect negative effects of roadways are identified among the leading range‐wide threats to many pit vipers in North America (Petersen and Sealy 2021). While direct road mortality is the most visible, easily measured, and frequently cited negative consequence of road interactions for pit vipers and other snakes (Gibson and Merkle 2004; Andrews and Gibbons 2005; Hartmann et al. 2011), there is also evidence of sub‐lethal road effects on snake behavior. Andrews and Gibbons (2005) found variation in road crossing propensity and speed across snake species, body size classes, and specific road features (road width and surface type), while others have provided strong evidence for road avoidance through longitudinal monitoring of movement, road interactions, and comparisons with random walk simulations (Robson and Blouin‐Demers 2013; Siers et al. 2014). Further, Shine et al. (2004) found that Garter Snakes ( *Thamnophis sirtalis* ) were less effective at chemosensory tracking females across road surfaces relative to natural land covers. For snake species that exhibit prolonged mate searching by males, this chemosensory disruption by roadways could carry significant fitness consequences in the form of elevated travel costs and/or reduced mate location success (Madsen and Shine 1993; Shine et al. 2004; Petersen et al. 2019). Taken together, sex, seasonal motivational states, and relative road interaction frequencies can play an important role in how individuals respond to roadways and the subsequent consequences of those responses.

Our overarching goal was to leverage a combination of radio telemetry and accelerometry (DeSantis et al. 2020) to evaluate the effects of roadways on mate‐searching movements and mate location success in a Georgia, USA, population of Timber Rattlesnakes ( *Crotalus horridus* ), a widespread mesopredator of southeastern forests that is threatened across much of its range, including recent documentation of road‐associated declines in the southeast (Jenkins et al. 2021). Using the same integrated data collection approach with this *C. horridus* population, Tipton et al. (2023) reported a positive association between road interactions and *C. horridus* movement distances and durations. However, the important roles of sex and behavioral season were not considered in that analysis because of sampling limitations. Although 26 individuals were included for analysis in the previous study, within‐group sampling by factor levels such as sex and behavioral season significantly reduced the power of the analysis. Therefore, after increasing the sample nearly twofold, additional analyses were made possible for the current study. Here, we address the following questions and associated predictions: (1) How do sex and behavioral season influence the association between road interactions and patterns of movement? We predicted that male and female movement measures would exhibit seasonal patterns associated with a prolonged male search‐based mating system, with males increasing movement during the mating season and females displaying no seasonal shifts. However, we also expected male and female rattlesnakes interacting with roadways to exhibit greater measures of movement across seasons relative to rattlesnakes not interacting with roads (Tipton et al. 2023). (2) Given the above predictions, do rattlesnakes interact with roads more frequently during mating periods relative to non‐mating periods of the active season? We predicted that male rattlesnakes would interact with roads (number of road crossing events and relocations < 25 m from a roadway) more during the mating season as a result of elevated movement in mate‐searching efforts (Waldron et al. 2006), while female road interaction count would show no seasonal differences (DeSantis et al. 2019). (3) Lastly, how do road interactions during the mating season relate to mate location success? We predicted that road interactions for male and female *C. horridus* would be negatively associated with mate location success, in line with the road avoidance (Robson and Blouin‐Demers 2013; Siers et al. 2014) and chemosensory disruption hypotheses (Shine et al. 2004).

## 2 Materials and Methods

### 2.1 Study Site and Study Species

The field study site is located in Putnam County, Georgia, within the Cedar Creek Wildlife Management Area (CCWMA) and Oconee National Forest (ONF) (centered on N 33°14′16.33″ W 83°30′48.24″). The site is characterized by high levels of heterogeneity, with managed forests, clear cuts, scattered residential properties, and an abundance of paved and unpaved roads that rattlesnakes have been observed to cross (Tipton et al. 2023). The paved roads are moderately trafficked and are eight meters wide. Two paved roads transect areas of the site containing rattlesnakes included in this study, and each have an approximate traffic volume of 122 vehicles per day (Georgia Department of Transportation 2022). The unpaved dirt and gravel roads at the site vary between approximately six and eight meters wide. These roads experience low rates of vehicular use with estimated traffic volumes not typically exceeding 20 vehicles per day (personal observations). Accordingly, this system is ideal for assessing the effects of roads on snake movement behavior (Tipton et al. 2023).

Timber Rattlesnakes ( *C. horridus* ) are large‐bodied pit vipers historically distributed across eastern North America (Martin et al. 2008). Although locally abundant at our study site, a recent conservation action plan for *C. horridus* described a patchy distribution and declining trend for populations in the Piedmont ecoregion of Georgia (Jenkins et al. 2021). That same report implicates roads among the primary range‐wide threats, and previous work demonstrates the damaging impacts of roads in northern *C. horridus* populations through direct mortality, interrupted dispersal, habitat fragmentation, and reduced gene flow (Clark et al. 2010; Martin et al. 2021). These effects have been linked to local extirpations, and remaining northern populations are of significant conservation concern (Martin et al. 2021). However, despite similar reports of population declines outside northern populations, *C. horridus* is only formally protected in 12 of the 30 states within its distribution (Martin et al. 2021). Given the above‐documented role of roads in extirpations of northern populations of *C. horridus* , it is prudent to improve our understanding of road effects in the southeast to proactively inform conservation initiatives.

### 2.2 Field Data Collection

Radio telemetry and accelerometry monitoring of *C. horridus* at this site began in June 2020 and ran through October 2023 for this study. Adult rattlesnakes were found through targeted visual encounter surveys or opportunistically collected at the study site and transported back to an approved lab space at Georgia College & State University. Collected individuals were sexed using a probe, and snout‐vent length (SVL) (mm), tail length (mm), and mass (g) were recorded to identify reproductively mature adults (SVL > 90 cm) following Gibbons (1972). Following isoflurane anesthesia using the open‐drop method, radio transmitters (Holohil Systems Ltd., Model SB‐2) and accelerometers (Technosmart Europe srl., AXY‐5) were internally implanted (Reinert and Cundall 1982) and sutured to a rib (Hardy and Greene 1999, 2000) following previously established protocols (DeSantis et al. 2020). Implants (radio transmitter = 5 g; accelerometer = 8 g) were ≤ 3% of each individual's body mass at the time of each procedure. Radio transmitters had a battery life of 10–12 months, and accelerometers recorded for up to 12 months at the selected recording frequency of 1 Hz. Accelerometers also housed a temperature sensor that continuously recorded internal body temperature for the entire battery life. Following device implantation, rattlesnakes were typically released at the original site of capture within three to four days of the procedure (during normally scheduled data collection visits). In some rare cases, rattlesnakes were held for up to one week post‐implantation to avoid releases during environmental conditions that could hinder normal recovery (unseasonably low temperatures in spring or fall, or rainy conditions). Radio telemetry relocation occurred every three to four days across the entire active season (April–October). Additional observations were made daily or every other day during the mating seasons (August–October) to increase detection of reproductive pairings for mate location success quantification (see Section 2.5). These additional observations during the mating season were only to enhance detection of reproductive pairings and were not included in the calculation of movement metrics across behavioral seasons (i.e., movement was estimated in the same manner [using two weekly relocation points] across all data). Relocations during the winter inactive period (November–March) occurred biweekly, but these data were not included in any analyses for this study. Detailed behavioral observations were recorded during each relocation along with environmental conditions (i.e., ambient temperature, weather, cloud cover), habitat type, and geographic coordinates using a hand‐held GPS (Garmin Oregon 700, accuracy ≤ 5 m). Individuals selected for device implantation were typically limited to two full active seasons of data collection (i.e., maximum of three surgical procedures over ca. 24 months), minimizing the negative effects of repeated implantation and extraction procedures.

### 2.3 Movement Quantification—Radio Telemetry

A series of spatial movement metrics were calculated for both non‐mating and mating seasons using geographic coordinate data collected during radio telemetry relocations. These measures included: meters per day (MPD), calculated as the distance between relocations divided by the number of days between relocations and then averaged; distance per movement (DPM), calculated as the mean straight‐line distance between relocations greater than 5m; and motion variance (MV) derived from dynamic Brownian bridge movement models (dBBMMs) (Kranstauber et al. 2012; Silva et al. 2018). Motion variance estimated the variance among movement distances within a moving window of nine radio telemetry relocations (i.e., one‐month increments of relocations). Higher MV values indicate more variable movement across the sampling duration considered (calculated within behavioral seasons, in this case). Home ranges were estimated with 100% minimum convex polygons (MCP); we opted for the MCP to maximize comparability with the companion study from this system (Tipton et al. 2023) as well as for the consistency of MCP home ranges relative to probabilistic estimators (Row and Blouin‐Demers 2006). Movement distance metrics were calculated from raw data in Microsoft Excel (version 16.78), MV was calculated in R (version 4.2.2, R Studio Team 2023.06.1+524) using the *move* package (version 4.1.10) (Kranstaube et al. 2018), and MCP home range sizes were calculated in R using the *adehabitatHR* package (version 0.4.20) (Calenge 2006; R Core Team 2023). All metrics were calculated and averaged within sex (female, male) and behavioral seasons (non‐mating, mating) for statistical analysis.

### 2.4 Activity Quantification—Accelerometry

General accelerometer data collection and processing protocols were developed and validated for rattlesnakes by DeSantis et al. (2020). To quantify activity patterns from acceleration data, we calculate dynamic body acceleration (DBA) metrics (Wilson et al. 2020) used for quantifying the energetics and overall intensity of animal movement (Qasem et al. 2012; Wilson et al. 2020). The suitability of these measures for quantifying activity in other squamates has been previously demonstrated (Ariano‐Sánchez et al. 2022). Using the *Tagtoools* package in R (R Core Team 2023; DeRuiter et al. 2024), DBA metrics were calculated over a 20‐s moving window and then averaged across 24 h to obtain mean daily activity intensity estimates across the non‐mating and mating seasons. The two DBA metrics calculated were overall dynamic body acceleration (ODBA) and vectorial dynamic body acceleration (VeDBA) (Qasem et al. 2012). ODBA is the absolute sum of the dynamic body acceleration (DBA) along the *x*, *y*, and *z* axes.

ODBA=∣DBA·x∣+∣DBA·y∣+∣DBA·z∣

While VeDBA is the vector of DBA along the *x*, *y*, and *z* axes.

$$\sqrt{\mathrm{DBA}\cdot x^{2}+\mathrm{DBA}\cdot y^{2}+\mathrm{DBA}\cdot z^{2}}$$

Both metrics were calculated and averaged within sex (female, male) and behavioral seasons (non‐mating, mating) for statistical analysis.

### 2.5 Mate Location Success

To explore associations between road interactions, movement, and mate location success, we devised a simple categorization scheme for mate location success based on field observations. Mating location success was defined as an observation of a radio‐tracked individual within 5 m of another adult *C. horridus* of the opposite sex (i.e., a potential mating partner) during the mating season (August to mid‐October). These observations did not necessarily need to include visual confirmation of coitus, as we were only interested in the relative ability of males to locate females and how this related to road interactions. A combination of visual indicators of conspecific sex was typically relied upon in these instances (i.e., when an untagged conspecific was detected in close proximity to a tagged rattlesnake). In all cases, direct observation of reproductive behavior (coitus or courtship) or visual cues like significant sexual dimorphism in total body size and tail length facilitated confident assessment of the sex of untagged rattlesnakes without needing to restrain them. For statistical analysis, we categorized individuals into two mate location success categories (mating binomial [MB]: MB 0 = not observed in a reproductive pairing, MB 1 = observed in at least one reproductive pairing), as total mate location successes within individuals only ranged from zero to three, with most individuals being observed in zero (39/62) or one (16/62) reproductive pairing. For mate location success analyses, we only included individuals that were subjected to radio telemetry and/or accelerometry data collection across an entire mating season.

### 2.6 Road Interactions

Using ArcGIS (ArcGIS Desktop 10.8.2), the straight‐line distance (m) to the nearest roadway was quantified for each relocation point within the non‐mating (April–July) and mating seasons (August–October) for every geographic coordinate set collected between June 2020 and November 2023. These distances enabled quantification of the number of road interactions within each individual, defined as relocations when rattlesnakes approached roadways within 25 m and/or crossed a road. The primary rationale for this 25 m threshold was to maintain continuity with the companion study's methods (Tipton et al. 2023), as the 25 m cutoff was based on the “goldilocks” zone identified by Tipton et al. 2023, where that threshold encompassed at least one relocation point preceding all road crossing events documented in the dataset (excluding outlying migratory road crossing events), meaning that the movement behavior during those timeframes was more likely to include behavior in close association with a roadway. Similar to mate location success, we categorized individual snake seasons into two road interaction categories (Road Interaction Binomial [RIB]: RIB 0 = did not interact with a road, RIB 1 = interacted with a road at least once) within behavioral seasons for statistical analysis.

### 2.7 Statistical Analyses

We used a linear mixed effects modeling (LME) framework to test our stated predictions regarding associations between movement behavior, road interactions, and mate location success. We first fit models to evaluate the effects of sex and season on the association between movement measures and road interactions. Individual response variables (radio telemetry movement and accelerometry activity metrics) were modeled separately to evaluate individual associations with the fixed effects of sex (male, female), behavioral season (non‐mating, mating), and road interaction category (RIB 0, 1) (Table 2). For accelerometer metric models (ODBA, VeDBA), we took advantage of the higher temporal resolution of these data to model the associations between activity level and road interactions at a daily scale (rather than averaging across broader time scales [i.e., season]). We adopted this fine‐scale approach to our accelerometer analysis based on the scale‐dependent associations between road interactions and movement identified by Tipton et al. (2023), where a significant increase in movement associated with road interactions was detected at the daily scale but not across the entire active season. Daily road interactions were determined using the same criteria as above, but with individual days serving as the categorization unit as opposed to seasons. This approach leverages continuous acceleration logging for a more direct evaluation of the associations between activity level and road interactions. These accelerometer models included the fixed effects of daily road interaction category (RIB 0, 1), sex, season, daily mean body temperature (derived from temperature loggers coupled to accelerometers), and snake body size (snout‐vent length) (Table 3). Next, a Fisher's Exact Test was used to determine whether the distribution of road interactions among male and female rattlesnakes differed across the non‐mating and mating seasons. We then fit models for individual response variables derived from means only calculated within mating seasons, and included the fixed effects of sex, road interaction category, and mate location success category (mating binomial [MB]: MB 0, MB 1) (Table 4). In all cases, a two‐stage stepwise model selection approach was employed by first running a model including all fixed effects of interest with only linear terms, then sequentially adding interaction terms between fixed effects in subsequent models, and ultimately comparing measures of model fit using the Akaike Information Criterion (AIC) (Tables A1 and A2). Given the more limited within‐group sampling for accelerometer data, and the need for two‐ and three‐way fixed effect interactions to facilitate testing of our hypotheses, we were unable to include the activity metrics (ODBA, VeDBA) in these mate location success models (Table 3). Snake ID and year were modeled as random effects in all models, controlling for data dependency across time. Radio telemetry spatial movement metric response variables included MPD, DPM, MV, and 100% MCP; accelerometer activity metric response variables included ODBA and VeDBA. All response variables were log‐transformed to achieve normality prior to analyses. After fitting individual LMEs, we calculated estimated marginal means (EMMs) to enable pairwise comparisons (Tables A3, A4, A5, A6, A7) between response variables across individual fixed effects factor levels (sex, season, RIB, MB). For all analyses, α was set at 0.05.

## 3 Results

### 3.1 Movement Quantification—Radio Telemetry

Between June 2020 and November 2023, 52 unique rattlesnakes were included in radio telemetry monitoring. Due to mortality events, equipment malfunctions, or female gravidity, 44 of the 52 individuals were included in statistical analyses (Female: *N* = 22, Male: *N* = 22). These 44 individuals contributed 104 behavioral seasons in radio telemetry data [Mating: *N* = 62 (Male: *N* = 30, Female: *N* = 32); Non‐mating: *N* = 42 (Male: *N* = 22, Female: *N* = 20)]. To control for repeated measures from individuals that contributed multiple seasons, individual ID and year were included as random effects during statistical analyses. Among these 44 individual rattlesnakes, 20 individuals (14 males, 6 females) interacted with a roadway at least once for a total of 87 confirmed road interactions used for placement into the road interaction categories. Sex and season‐specific mean (±SD) measures of MPD, DPM, MV, and 100% MCP are provided in Table 1.

**TABLE 1:** Mean ± standard deviation and range within sex (male, female) and behavioral season (non‐mating [April–July], mating [August–October]) for meters‐per‐day (MPD), distance‐per‐movement (DPM), motion variance (MV), 100% minimum convex polygons (MCP), overall dynamic body acceleration (OBDA), and vectorial dynamic body acceleration (VeDBA).

Movement metrics	Male non‐mating	Male mating	Female non‐mating	Female mating
MPD (m)	26.82 ± 14.24 (9.01–64.99)	44.78 ± 22.63 (12.29–86.11)	20.75 ± 8.68 (5.56–37.78)	20.57 ± 9.03 (6.51–44.85)
DPM (m)	122.71 ± 46.55 (46.09–245.84)	182.33 ± 90.33 (52.51–373.77)	91.11 ± 31.14 (22.02–140.15)	89.86 ± 38.28 (35.36–197.35)
MV	2.38 ± 2.03 (0.19–8.66)	8.12 ± 10.44 (0.17–47.48)	1.41 ± 1.24 (0.059–5.31)	1.18 ± 1.31 (0.07–6.62)
MCP (ha)	32.42 ± 30.07 (2.92–122.28)	32.72 ± 25.32 (0.99–98.71)	13.16 ± 9.22 (0.41–34.20)	10.08 ± 6.75 (0.88–27.74)
ODBA	0.020 ± 0.012 (0.005–0.075)	0.027 ± 0.022 (0.005–0.129)	0.014 ± 0.009 (0.005–0.072)	0.017 ± 0.010 (0.005–0.096)
VeDBA	0.015 ± 0.008 (0.004–0.053)	0.019 ± 0.015 (0.004–0.088)	0.012 ± 0.006 (0.004–0.049)	0.013 ± 0.007 (0.005–0.066)

### 3.2 Activity Quantification—Accelerometry

Of the 52 individuals included in radio telemetry monitoring, 37 were also equipped with accelerometers. Given unanticipated device failures and an uneven number of radio transmitters and accelerometers for deployment, not all individuals received an accelerometer in addition to a radio transmitter. Of the 44 individuals that contributed radio telemetry field data, 34 also contributed accelerometer data for statistical analyses (Female: *N* = 15, Male: *N* = 19). These 34 individuals contributed 44 behavioral seasons in accelerometer data [Mating: *N* = 26 (Male: *N* = 13, Female: *N* = 13); Non‐mating: *N* = 18 (Male: *N* = 10, Female: *N* = 8)]. Again, rattlesnake ID and year were included as random effects across all models to account for the repeated measures design. Sex and season‐specific mean (±SD) measures of ODBA and VeDBA are presented in Table 1.

### 3.3 Linear Mixed Effects Models: Radio Telemetry Seasonal Movement Metrics

**FIGURE 1:**
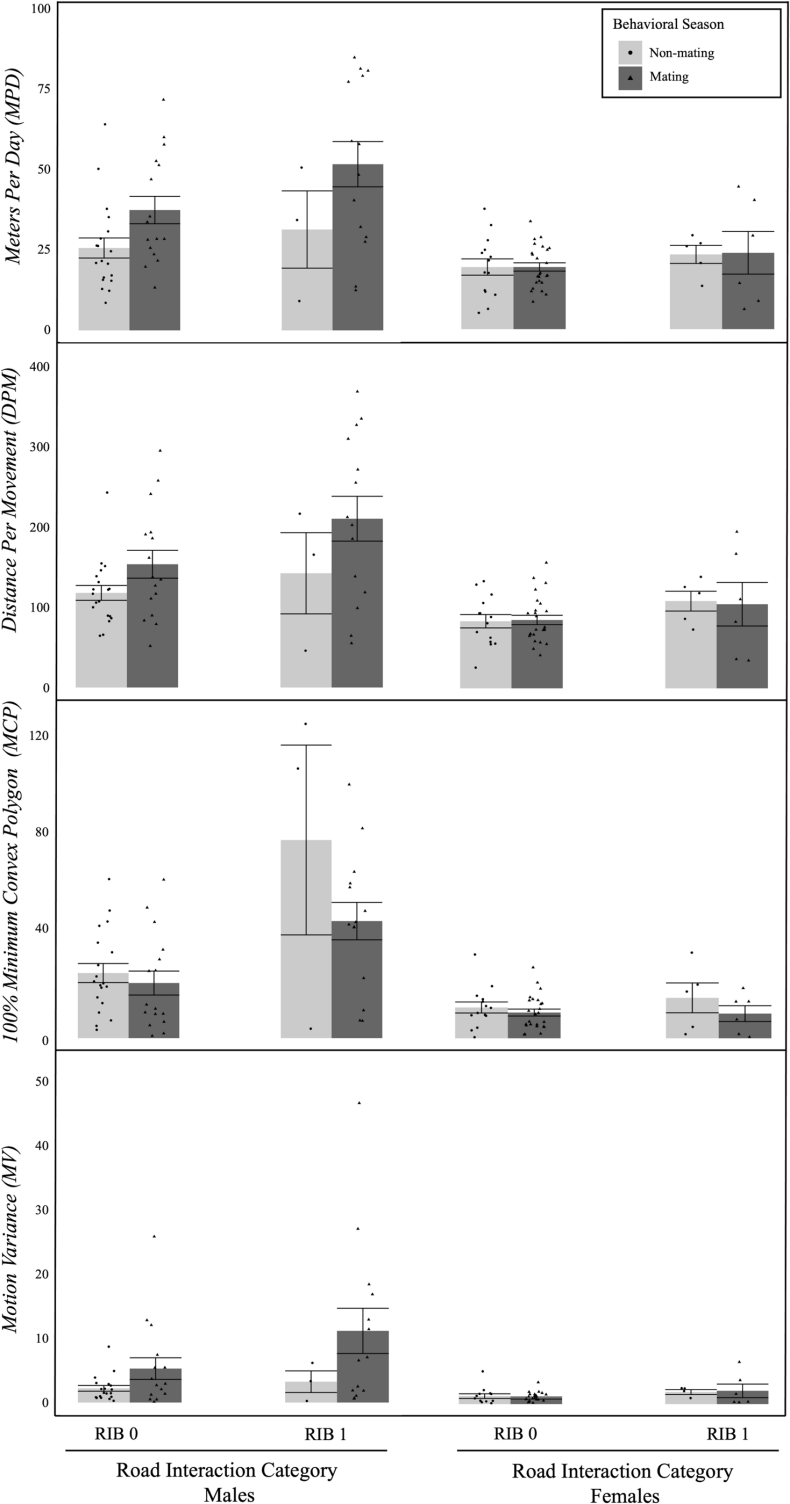
Mean (±SE) male and female seasonal movement and space‐use measures within behavioral season (Non‐Mating, Mating) and road interaction category (RIB 0 [no road interaction observed], RIB 1 [at least one road interaction observed]) across sampling years (2020–2023). Movement measures include meters per day, distance per movement, and motion variance. The space‐use measure includes 100% minimum convex polygon. Within‐group sample sizes: Male Non‐Mating RIB 0: 19; Male Mating RIB 0: 16; Male Non‐Mating RIB 1: 3; Male Mating RIB 1: 14; Female Non‐Mating RIB 0: 14; Female Mating RIB 0: 25; Female Non‐Mating RIB 1: 5; Female Mating RIB 1: 6. * denotes statistical significance for model parameter effects.

Radio telemetry seasonal movement LME models included sex, season, and road interactions (RIB) as fixed effects and MPD, DPM, MV, and 100% MCP as response variables. AIC values were comparable across most model structures (Table A1), but generally lowest for the models including only one interaction term between sex and season. There was a significant main effect of sex on MPD (Table 2), with males moving more than females overall. For MPD, there was also a significant interaction between sex and season (Table 2), indicating that males (*t*_67.2_ = 3.56, *p* = 0.001), but not females (*t*_65.8_ = 0.015, *p* = 0.99), increased MPD during the mating season relative to the non‐mating season (Figure 1). There were no significant effects of season or road interaction category on MPD. A significant main effect of sex was also detected in the DPM model (Table 2), with males again moving significantly more during the mating season relative to the non‐mating season (Figure 1). As in the MPD model, there were no significant effects of either road interactions or season on DPM. For both MV and 100% MCP there was again a significant main effect of sex (Table 2), as males moved more variably and occupied significantly larger home ranges than females. For MV, there was a marginal interaction between sex and season, showing that males, but not females, elevated motion variance during the mating season relative to the non‐mating season. However, this marginal sex and season interaction effect in MV was not maintained in the post hoc pairwise tests (*t*_69.5_ = 2.15, *p* = 0.07). No significant interactions between sex and season were found in the MCP model (Table 2; Figure 1), indicating that the increase in male movement during the mating season seen in MPD and DPM was not detected for home range size (*t*_63.3_ = −0.73, *p* = 0.72). For females, no differences were detected for either MV (*t*_67_ = −0.68, *p* = 0.75) or MCP (*t*_62.4_ = −0.59, *p* = 0.80). As for MPD and DPM, there were no detected main effects or interaction effects of road interactions (RIB) on MV or MCP.

**TABLE 2:** Coefficients, standard error (SE), and *p*‐values for individual radio telemetry‐derived seasonal movement and space‐use model parameters. Reference levels for categorical parameters are in parentheses [(Male), (Non‐mating), (RIB 1 = road interaction category “Yes”)], and coefficients can be used to interpret the direction of individual effects.

Model parameter	Coefficient	SE	*p*
**Meters per day (MPD)**			
Sex (male)	0.69	0.15	< 0.001*
Season (non‐mating)	−0.002	0.12	0.99
RIB (1)	0.13	0.11	0.26
Sex*Season	−0.44	0.18	0.02*
**Distance per movement (DPM)**			
Sex (male)	0.61	0.12	< 0.001*
Season (non‐mating)	0.02	0.101	0.84
RIB (1)	0.14	0.094	0.15
Sex*Season	−0.33	0.15	0.03*
**Motion variance (MV)**			
Sex (male)	1.42	0.304	< 0.001*
Season (non‐mating)	0.21	0.303	0.496
RIB (1)	0.3	0.26	0.197
Sex*Season	−0.85	0.43	0.05*
**100% Minimum convex polygon (MCP)**			
Sex (male)	0.67	0.29	0.03*
Season (non‐mating)	0.0002	0.29	0.99
RIB (1)	0.22	0.15	0.15
Sex*Season	0.73	0.4	0.07

* denotes statistical significance for model parameter effects.

For all of the above models, the results from post hoc pairwise tests are summarized in the Appendix (Table A3).

### 3.4 Linear Mixed Effects Models: Accelerometer Fine‐Scale Activity Metrics

LME models included sex, season, road interactions (RIB), snout‐vent length (SVL), and mean daily body temperature as fixed effects and ODBA and VeDBA as response variables (Table 3). A significant main effect of body temperature was detected in both models (Table 3). A marginal effect of sex was also detected in both models (Table 3), with males exhibiting slightly larger measures of activity than females monitored. In the ODBA model, there was a significant interaction between sex and RIB (Table 3), with male ODBA exhibiting a positive association with days that included a road interaction (*t*_809_ = −2.204, *p* = 0.05), and females displaying no association between ODBA and road interactions (*t*_807_ = 0.804, *p* = 0.57). In the VeDBA model, there was also a significant interaction between sex and RIB (Table 3) with male VeDBA exhibiting a marginal positive association on days with road interactions (*t*_809_ = −2.18, *p* = 0.05), and females displaying no association between VeDBA and road interactions (*t*_807_ = 0.823, *p* = 0.65). There were no main effects of season, SVL, or RIB on either the ODBA or VeDBA models. Post hoc estimated marginal means pairwise comparisons conducted on the ODBA model detected a significant difference within the group of individuals that interacted with roads, between males and females (*t*_102.3_ = −2.76, *p* = 0.0122). The same significant difference in this group, between males and females (*t*_101.1_ = −2.732, *p* = 0.0132), was detected in post hoc estimated marginal means pairwise comparisons from the VeDBA model.

**TABLE 3:** Coefficients, standard error (SE), and *p*‐values for individual seasonal accelerometer‐derived activity intensity (overall dynamic body acceleration [ODBA], vectorial dynamic body acceleration [VeDBA]) and road interaction (RIB) model parameters. Reference levels for categorical parameters are in parentheses [(male), (non‐mating)], and coefficients can be used to interpret the direction of individual effects.

Model parameter	Coefficient	SE	*p*
**ODBA**			
Sex (male)	0.66	0.34	0.07
RIB (0)	−0.22	0.28	0.42
SVL	0.001	0.001	0.34
Body temperature	0.26	0.07	< 0.001*
Season (non‐mating)	0.04	0.07	0.59
Sex*RIB	0.68	0.35	0.05*
**VeDBA**			
Sex (male)	0.65	0.35	0.08
RIB (0)	−0.23	0.28	0.41
SVL	0.001	0.001	0.41
Body temperature	0.26	0.07	< 0.001*
Season (non‐mating)	0.03	0.07	0.67
Sex*RIB	0.68	0.35	0.05*

* denotes statistical significance for model parameter effects.

### 3.5 Fisher's Exact Test: Seasonal Road Interactions

A Fisher's Exact Test revealed an association between behavioral season and road interactions for males, with most road interactions occurring during the mating season (*p* = 0.02). There was no association between behavioral season and road interactions for females (*p* = 0.73). This is also clearly seen in the raw count data, as 14 out of 31 monitored males interacted with roads at least once (31 total interactions) during the mating seasons of 2020–2023, whereas only three out of 22 monitored males interacted with roads at least once (19 total interactions, 13 from one individual) during the non‐mating seasons of 2020–2023. Conversely, only six out of 32 monitored females interacted with roads at least once (12 total interactions) during the mating seasons of 2020–2023, and only five out of 19 monitored females interacted with roads (16 total interactions) during the non‐mating seasons of 2020–2023.

### 3.6 Fisher's Exact Test: Mate Location Success

Males placed in either road interaction category were no more or less likely to be placed in either mate location category (*p* = 0.46). Of the 14 males that interacted with roads during mating seasons, four were observed in a reproductive pairing. Of the 16 males that did not interact with roads, seven were observed in a reproductive pairing. There was also no significant association between placement in road interaction and mate location categories for females (*p* = 1). Of the six females that interacted with roads during mating seasons within the study period, two were observed in a reproductive pairing. Of the 26 females that did not interact with roads, 10 were observed in a reproductive pairing.

### 3.7 Linear Mixed Effects Models: Mate Searching and Mate Location Success

**FIGURE 2:**
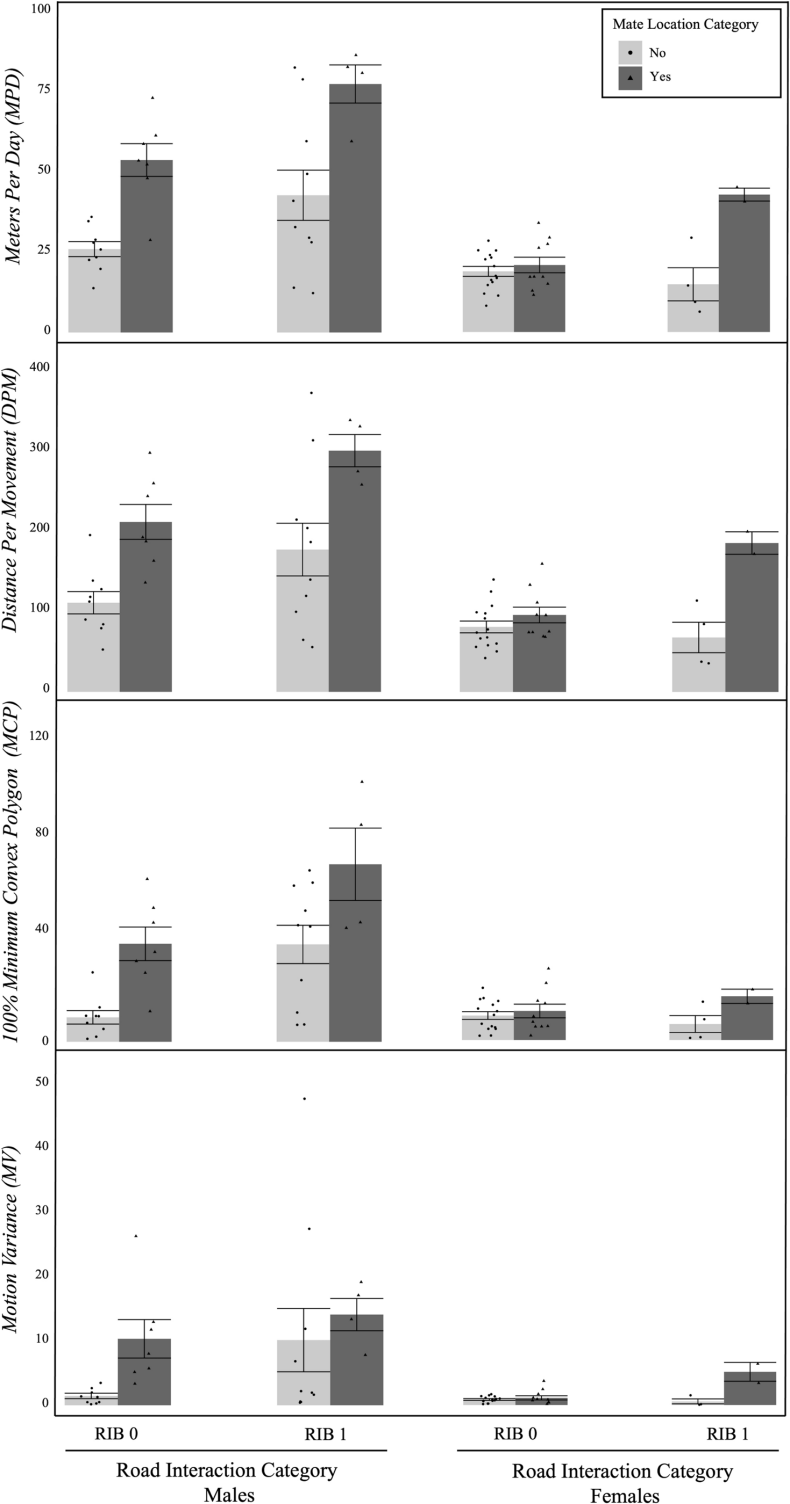
Mean (±SE) male and female seasonal movement and space‐use measures within mate location success (MB 0 [No], MB 1 [Yes]) and road interaction categories (RIB 0 [no road interaction observed], RIB 1 [at least one road interaction observed]) across sampling years (2020–2023). Movement measures include meters per day, distance per movement, and motion variance. The space‐use measure includes 100% minimum convex polygon (MCP). Within‐group sample sizes: Male MB 0 RIB 0: 9; Male MB 1 RIB 0: 7; Male MB 0 RIB 1: 10; Male MB 1 RIB 1: 4; Female MB 0 RIB 0: 15; Female MB 1 RIB 0: 10; Female MB 0 RIB 1: 4; Female MB 1 RIB 1: 2.

Mate location success LME models included sex, mate location (MB), and road interaction categories (RIB) as fixed effects and MPD, DPM, 100% MCP, and MV as response variables (Table 4). AIC values were comparable across most model structures (Table A1), but generally lowest for the models including interaction terms between all fixed effects. The only main effect detected across all models was one of sex on MV (Table 4). For the movement estimates, MPD and DPM, there were significant interactions detected between all fixed effects. The interaction between sex and mate location indicates that male, but not female, movement [MPD and DPM; (Table 4)] was positively associated with mate location success (Figure 2). Post hoc estimated marginal means pairwise comparisons corroborated this trend for MPD (males: *t*_45.4_ = −3.99, *p* = 0.0005, females: *t*_49.9_ = −1.78, *p* = 0.15) and also for DPM (males: *t*_47.9_ = −3.52, *p* = 0.002, females: *t*_51_ = −1.572, *p* = 0.23). The interaction between sex and RIB indicates that male movement [MPD and DPM; (Table 4)] was positively associated with road interactions while female movement was not (Figure 2). The post hoc pairwise comparisons for the factor levels involved in this interaction did not align with the model results for male MPD (*t*_47.3_ = −1.54, *p* = 0.24) and DPM (*t*_43.9_ = −1.72, *p* = 0.18), but the involvement of these variables in a significant three‐way interaction likely makes these two‐way interaction contrasts misleading (see three‐way interaction results, below). The pairwise comparisons do, however, mirror the model result for female movement not correlating with road interactions [MPD (*t*_50.3_ = −0.997, *p* = 0.54) and DPM (*t*_50.1_ = −1.101, *p* = 0.47)]. For individuals that did not interact with roads, movement [MPD and DPM (Table 4)] was not associated with mate location success (i.e., placement in MB 1) (MPD: *t*_52.8_ = −2.138, *p* = 0.07; DPM: *t*_51.2_ = −1.960, *p* = 0.11). Converesely, for individuals that did interact with roads, there was a positive association between movement (MPD and DPM) and mate location success (MPD: *t*_52.7_ = −3.213, *p* = 0.005; DPM: *t*_52.1_ = −2.180, *p* = 0.01; Figure 2). However, the presence of a significant three‐way interaction between sex, mate location, and road interactions (Table 4) make the above two‐way interactions misleading. This three‐way interaction indicates that male movement (MPD and DPM) was positively associated with mate location success among males that did not interact with roads (MPD: *t*_49.8_ = −3.43, *p* = 0.005; DPM: *t*_50.6_ = −3.251, *p* = 0.008), but not for males that did interact with roads (MPD: *t*_43.8_ = −2.721, *p* = 0.11; DPM: *t*_46.5_ = −1.851, *p* = 0.25). Meanwhile, there were no associations between movement (MPD and DPM) or mate location success for females in either road interaction category [MPD: (RIB 0: (*t*_47.4_ = 0.396, *p* = 0.99); RIB 1: (*t*_52.1_ = −2.383, *p* = 0.08)); DPM: (RIB 0: (*t*_45.7_ = 0.515, *p* = 0.97); RIB 1: (*t*_51.9_ = −2.182, *p* = 0.13))]. These results were further supported by subsequent Wilcoxon Rank Sum Tests performed to compare the total number of mating partners acquired, within sexes, between road interaction categories (Males: W = 11.5, *p* = 0.67; Females: W = 12, *p* = 0.62).

**TABLE 4:** Coefficients, standard error, and *p*‐values for individual mating season (August–October) movement and home range metric models. Fixed effects included sex, road interaction category (RIB), and mate location success category (MB), with reference levels for each in parentheses (Male, MB 0, RIB 0). Coefficients can be used to interpret the direction of individual main effects. For brevity, the factor level contrasts (interpreted from post hoc estimated marginal means tests) for the significant two‐ and three‐way interactions can be referenced in the results text or in the corresponding tables in the Appendix.

Model parameter	Coefficient	SE	*p*
**Meters per day**			
Sex (male)	0.296	0.18	0.09
MB (0)	−0.079	0.19	0.67
RIB (0)	−0.29	0.22	0.18
Sex*MB	0.795	0.27	0.004*
Sex*RIB	0.63	0.28	0.03*
MB*RIB	0.97	0.38	0.01*
Sex*MB*RIB	−1.14	0.49	0.03*
**Distance per movement**			
Sex (male)	0.25	0.16	0.13
MB (0)	−0.09	0.17	0.59
RIB (0)	−0.225	0.201	0.27
Sex*MB	0.72	0.24	0.005*
Sex*RIB	0.59	0.26	0.03*
MB*RIB	0.84	0.35	0.02*
Sex*MB*RIB	−1.05	0.46	0.03*
**Motion variance**			
Sex (male)	0.67	0.31	0.04*
MB (0)	−0.16	0.37	0.67
RIB (0)	0.15	0.390	0.70
Sex*MB	2.12	0.54	< 0.001*
Sex*RIB	0.51	0.504	0.32
MB*RIB	1.497	0.896	0.09
Sex*MB*RIB	−2.38	1.13	0.04*
**100% Minimum convex polygon**			
Sex (male)	−0.32	0.35	0.37
MB (0)	−0.26	0.38	0.49
RIB (0)	−0.66	0.44	0.14
Sex*MB	1.87	0.54	0.001*
Sex*RIB	2.24	0.58	< 0.001*
MB*RIB	1.36	0.77	0.08
Sex*MB*RIB	−2.59	1.007	0.01*

* denotes statistical significance for model parameter effects.

The same two‐way interactions seen for MPD and DPM were maintained for the home range estimate (MCP), except for the interaction between mate location (MB) and road interaction (RIB). However, because the same three‐way interaction (sex*MB*RIB) seen in the movement (MPD and DPM) models was also maintained in the MCP model (Table 4), the results from the post hoc pairwise comparisons of the two‐way interactions cannot be taken alone. The two‐way interaction between sex and MB indicates that male home range size (MCP), in addition to movement (MPD and DPM), was positively associated with mate location success, while female home range size (MCP) was not (Table 4; Figure 2). Post hoc estimated marginal means pairwise comparisons of the MCP model do not support this trend for males (*t*_49.7_ = −1.693, *p* = 0.183), but uphold the trend for females (*t*_43.6_ = −1.329, *p* = 0.342). This seems counterintuitive for the males until road interactions are considered. The two‐way interaction between sex and road interactions indicates that male MCP was positively associated with road interactions, while female MCP was not (Table 4). Post hoc estimated means pairwise comparisons conducted for this interaction again do not align with signals from the LME models for males (*t*_36_ = −1.59, *p* = 0.225) but do for females (*t*_49.4_ = 0.111, *p* = 0.992). As for MPD and DPM, the post hoc comparisons garnered from the three‐way interaction factor levels are the most informative. Male MCP is positively associated with mate location success for males without road interactions (*t*_50.3_ = −3.79, *p* = 0.0016), but not for males with road interactions (*t*_44.9_ = −0.754, *p* = 0.91). Female MCP was not associated with mate location success for those with (*t*_47_ = 0.637, *p* = 0.95) or without road interactions (*t*_52.2_ = −1.45, *p* = 0.48).

For MV, the above‐stated main effect of sex indicates that males moved more variably than females during the mating season (Table 4). A significant two‐way interaction between sex and mate location (Table 4) indicates that male MV was positively associated with mate location success (t_49.7_ = −3.95, *p* = 0.0005) while female MV was not (t_51_ = −1.334, *p* = 0.34). A significant three‐way interaction mirrors the relationship seen in MPD, DPM, and MCP (Figure 2). This interaction between sex, mate location success, and road interactions (Table 4) indicates that male MV was positively associated with mate location success for males without road interactions (*t*_50.8_ = −4.051, *p* = 0.0007), but not for males with road interactions (*t*_48.5_ = − 1.76, *p* = 0.29). No associations were observed for females [RIB 0 (*t*_44.2_ = 0.891, *p* = 0.84); RIB 1 (*t*_52.4_ = −2.076, *p* = 0.16)].

For all of the above models, the results from post hoc pairwise tests are summarized in the Appendix (Tables A4, A5, A6, A7).

## 4 Discussion

Using longitudinal monitoring of movement behavior in Timber Rattlesnakes ( *C. horridus* ) across several years, we aimed to supplement the currently limited body of knowledge regarding associations between roads and snake movement behavior. Specifically, we sought to identify whether road interaction frequency differed relative to sex and behavioral season and if sub‐lethal road interactions were associated with variation in movement patterns and observed mate location success. Below, we summarize key results in the context of each of our predictions and present potential mechanisms that might be underlying observed associations that could lead to the development of more targeted future studies or experiments.

### 4.1 Seasonal Movement Behavior and Road Interactions

Typical of a prolonged male mate‐searching mating system, males displayed significant increases in movement distances and motion variance during the mating season and also exhibited larger measures of movement (MPD, DPM) and home ranges (MCP) relative to females (Duvall et al. 1993; Madsen et al. 1993; Clark et al. 2014; DeSantis et al. 2019). In contrast, females displayed no seasonal differences in movement behavior. However, contrary to our predictions, the association between road interactions and movement does not change across behavioral seasons. Previous evidence from this site suggests that responses to roadways might only be detected at fine scales (Tipton et al. 2023). By leveraging high‐resolution accelerometer activity metrics (ODBA, VeDBA) for a more targeted evaluation of the association between activity and road interactions, we found that males displayed significantly greater levels of activity on days with road interactions compared to days without them (across seasons). Because days (within individuals) were classified into our road interaction categories for this analysis, rather than individuals being grouped into the categories as in our spatial movement models, these fine‐scale model results more directly associate road interactions with observed movement patterns. These findings generally reflect those presented in Tipton et al. (2023) and demonstrate the same trend of hidden variation in snake movement behavior being revealed by the high‐resolution logging of accelerometry. It should be emphasized that these associative analyses do not necessarily demonstrate a causal relationship between road interactions and movement, as days with elevated activity or movement might simply be associated with an increased probability of road encounters. More targeted experiments that facilitate road interactions and the subsequent behavioral responses in free‐ranging individuals could improve our ability to link road interactions to movement in this and other systems. Additionally, the more limited sample of accelerometer‐tagged snakes across mate location success categories precluded our ability to model the associations between activity level, road interactions, and mate location success. Nevertheless, the finding that males displayed a higher activity level on days with road interactions is particularly important to consider when evaluating the below‐reviewed associations between movement, road interactions, and mate location success.

### 4.2 Seasonal Shifts in Road Interaction Frequency

As predicted, a Fisher's Exact test showed that male rattlesnakes interacted with roads at a significantly higher rate during the mating season relative to the non‐mating season. The increase in road interactions by males during the mating season corresponds with the significant increase in movement expected from mate‐searching movements by males (DeSantis et al. 2019). This is a notable finding, as elevated road interactions likely carry elevated risks for males that are already experiencing greater costs from increased movement in mate‐searching efforts (Duvall et al. 1993). Also, as predicted, there were no differences in female road interactions across behavioral seasons, and females interacted with roads far less often than males. The limited total sample of female road interactions is most parsimoniously explained by reduced female movement and home range size relative to males across seasons, which is characteristic of many rattlesnake species (DeSantis et al. 2019). However, additional sampling or comparisons with random walk simulations (Robson and Blouin‐Demers 2013) could explore whether females exhibit behavioral avoidance of roads, which would carry important implications for mate‐searching males that interact with roads.

### 4.3 Road Interactions and Mate Location Success

Shine et al. (2004) found that male Garter Snakes ( *Thamnophis sirtalis* ) were less effective at locating females after crossing roadways and suggested that more rapid degradation of chemical cues on roadways likely inhibited male chemosensory trailing of females (Shine et al. 2004). Additionally, Tipton et al. (2023) found that *C. horridus* at the same site of the present study significantly elevated movement and activity during road interactions and also exhibited an increase in the distance to the nearest road immediately following road interactions (possibly indicative of a road avoidance response). Based upon this evidence from prior work, we predicted that these factors would drive a negative association between road interactions and mate location success for male and female *C. horridus* . However, our results did not support this prediction. The Fisher Exact Test indicated that, for both males and females, road interactions were not associated with mate location. Further, to ensure that our coarse categorization scheme for mate location was not overlooking a correlation between road interactions and the total number of unique mating pairings per individual, we performed subsequent Wilcoxon Rank Sum Tests to compare the number of mating partners observed for each individual, within sexes, between road interaction categories. Once again, these tests indicated no differences in mate location success by males and females between road interaction categories. There were also no significant main associations between mate location success, road interactions, and movement metrics across models (Table 4). However, significant two‐ and three‐way interactions in these models indicated differing associations between movement and the fixed effects factor levels (Table 4). Most notably, males that did not interact with roads during the mating season exhibited a significant positive association between all measures of movement and mate location success. This aligns with a male search‐based mating system, where male movement is the primary determinant of male reproductive success (Duvall et al. 1993; Madsen et al. 1993; Clark et al. 2014; DeSantis et al. 2019). Conversely, for males that interacted with roads during the mating season, increased movement was not associated with increased mate location success. While these results could be interpreted as reduced mate‐searching efficiency by road‐interacting males, the small within‐group sample sizes included in these two‐ and three‐way interactions do draw caution during interpretation (see Figure 2 caption). As was also the case for the daily activity models, the most parsimonious explanation for these results is simply that increased movement among males is positively associated with road interactions, and additional sampling plus an improved ability to measure reproductive success would allow a more effective analysis of the possible effects of road interactions on mate location.

## 5 Conclusions and Future Directions

We found strong evidence of increased road interactions by males during mating seasons and weaker evidence that might warrant future investigation into the possibility of reduced mate‐searching efficiency by males interacting with roads. While sexual selection is expected to favor increased male movement during mating periods in prolonged male search mating systems (Duvall et al. 1993; DeSantis et al. 2019), the elevated costs from road encounters (on top of natural selective pressures) carry important conservation implications (Clark et al. 2010). Although a negative association between road interactions and mate location success was not found here, the increase in male road interaction frequency during the mating season, along with the fact that eight of the 14 road‐interacting males in this study also crossed roads during the mating season, highlights the elevated risks incurred by these rattlesnakes. In line with this, the lone road mortality documented among our longitudinal sample was an adult male during the mating season (early October) of 2023. These demographic trends have been well established through previous observational studies of snake road mortality (Rosen and Lowe 1994; Bonnet et al. 1999) and seasonal variation in rattlesnake road mortality specifically (Aldridge and Brown 1995; Sealy 2002; Shepard, Dreslik, et al. 2008; Shepard, Kuhns, et al. 2008; Clark et al. 2010). However, this study is the first (to the best of our knowledge) to directly analyze the association between variation in movement and road interactions among longitudinally monitored rattlesnakes.

While the results do not support our predictions for reduced mate location success among road‐interacting rattlesnakes, future investigations that account for the methodological limitations in the present study could more effectively study these relationships. As previously stated, we opted to maintain the simple threshold (< 25 m) classification scheme for identifying road interactions for consistency with the companion study from this site (Tipton et al. 2023). However, future studies could evaluate the effectiveness of more probabilistic approaches for capturing relevant variation in movement behavior in association with roads, such as using contours around movement pathways from dBBMM utilization distributions for identifying possible road interactions, as was demonstrated by Jones et al. (2022) with King Cobras ( *Ophiophagus hannah* ). However, it should be noted that lag time between radio telemetry relocations is a key consideration when producing these movement pathway contours, and coarser tracking schedules (such as the one adopted for this study) are associated with higher % error in pathway estimation (Silva et al. 2020). The limited number of female road interactions observed also warrants future investigation, although reduced movement and space use relative to males likely best explain this result, rather than potential female road avoidance. Female snakes (including rattlesnakes) have been hypothesized to facilitate mate location through increased movement and chemical signaling via lipid‐based pheromones (Emlen and Oring 1977; Aldridge and Duvall 2002; Jellen and Aldridge 2014; Fromhage et al. 2016). While there was no association between movement and mate location for females, the two females that both interacted with roads and were observed in a reproductive pairing during our study displayed the largest mean measures of movement among all monitored females. Broadly, we feel that the possible role of female movement in rattlesnake (and other snake) mating systems is understudied and worth future attention. As increasingly sophisticated machine learning models are developed for accurate classification of distinct behavioral states in snakes (Hanscom et al. 2023), eventual remote and continuous monitoring of reproductive behavior could dramatically improve the ability to identify links between variation in movement behavior and reproductive outcomes. Lastly, while we originally set out to account for road surface type (paved, unpaved) in our analyses, we were unable to fit models that included additional factor level partitioning of already limited road interaction observations. Of the 87 road interactions included in analyses, 47 were interactions with unpaved (dirt/gravel) roads and 40 were interactions with paved roads. Future investigations could involve additional sampling of road interactions across road surface types or consider experimentally staging such interactions to increase observations.

Despite the various limitations and unanswered questions outlined above, our results can provide additional guidance for the conservation and management of imperiled pit viper populations exposed to roadways. Given that many pit vipers simultaneously serve as top predators and important prey species (Nowak et al. 2008), they can also represent useful biotic indicators of the extent to which roadways impact ecological communities (Carignan and Villard 2002; Sergio et al. 2008; Beaupre and Douglas 2011). For *C. horridus* specifically, roads are recognized as a leading range‐wide threat to population persistence, and populations across the piedmont region of Georgia are observed to be in decline (Jenkins et al. 2021; Martin et al. 2021). We recommend that, when possible, public recreation lands harboring threatened *C. horridus* populations reduce the number of roads transecting contiguous patches of forest. Even lower‐trafficked and unpaved roads, such as many found at our study site, could elevate human–rattlesnake conflicts or present sub‐lethal disruptions to rattlesnake movement behavior. Additionally, given the significant increase in road interactions for males during the mating season, a combination of educational outreach, signage, or seasonal road closures in public lands, as has been implemented at “snake road” in Illinois (Palis 2016), could help to mitigate negative impacts of roadways on rattlesnake populations.

## Author Contributions

**Elizabeth J. Noble:** data curation (equal), formal analysis (lead), funding acquisition (equal), investigation (equal), writing – original draft (lead), writing – review and editing (equal). **Anna F. Tipton:** data curation (equal), formal analysis (equal), funding acquisition (equal), investigation (equal), writing – review and editing (equal). **Morgan L. Thompson:** data curation (equal), investigation (equal), writing – review and editing (supporting). **John R. Powers:** data curation (equal), funding acquisition (equal), investigation (equal), writing – review and editing (supporting). **Amber A. Stubbs:** data curation (equal), investigation (equal), writing – review and editing (supporting). **William L. Tillett II:** data curation (equal), investigation (equal), writing – review and editing (supporting). **Jorge A. Vázquez Diosdado:** data curation (supporting), formal analysis (supporting), methodology (supporting). **Dominic L. DeSantis:** conceptualization (lead), data curation (equal), formal analysis (lead), funding acquisition (lead), investigation (equal), methodology (lead), resources (lead), supervision (lead), validation (lead), writing – original draft (lead), writing – review and editing (lead).

## Conflicts of Interest

The authors declare no conflicts of interest.

## Data Availability

The data and code that support the findings of this study are openly available through ResearchGate. Mate Location Dataset: https://www.researchgate.net/publication/385429512_Dataset_Seasonal_Movement_Road‐Interaction_Mate‐Location_Metrics_CRHO_2020‐2023. Activity Dataset: https://www.researchgate.net/publication/385429512_Dataset_Seasonal_Movement_Road‐Interaction_Mate‐Location_Metrics_CRHO_2020‐2023. Movement Dataset and Code: https://www.researchgate.net/publication/385428801_Rattlesnake_Movement_Analysis_Code_and_Data.

## Appendix

**TABLE A1:** Akaike Information Criterion (AIC) model fit scores for sex and season linear mixed effects models and mate location success models. Sex and season models included sex (male, female), season (mating, non‐mating), and road interaction category (RIB) as fixed effects. Mate location success models included sex (male, female), mate location category (MB), and road interaction category (RIB) as fixed effects. Both sets of models included meters‐per‐day (MPD), distance‐per‐movement (DPM), motion variance (MV), and 100% minimum convex polygon (MCP) as response variables.

Sex and season models	AIC	Mate location success models	AIC
**MPD Models**			
MPD ~ sex + season + RIB	169.45	MPD ~ sex + MB + RIB	92.12
MPD ~ sex * season + RIB	167.18	MPD ~ sex * RIB + MB	94.45
MPD ~ sex * season + sex * RIB	170.29	MPD ~ sex + RIB * MB	92.75
MPD ~ sex + season * RIB	172.46	MPD ~ sex * MB + RIB	90.74
MPD ~ sex * RIB + season	172.36	MPD ~ sex * MB + sex * RIB	92.74
MPD ~ sex * season * RIB	174.96	MPD ~ sex * MB * RIB	90.89
**DPM Models**			
DPM ~ sex + season + RIB	130.90	DPM ~ sex + MB + RIB	83.66
DPM ~ sex * season + RIB	130.25	DPM ~ sex * RIB + MB	86.24
DPM ~ sex * season + sex * RIB	133.79	DPM ~ sex + RIB * MB	85.03
DPM ~ sex + season * RIB	134.40	DPM ~ sex * MB + RIB	82.88
DPM ~ sex * RIB + season	134.31	DPM ~ sex * MB + sex * RIB	84.98
DPM ~ sex * season * RIB	139.34	DPM ~ sex * MB * RIB	83.82
**MV Models**			
MV ~ sex + season + RIB	325.16	MV ~ sex + MB + RIB	199.53
MV ~ sex * season + RIB	323.17	MV ~ sex * RIB + MB	199.20
MV ~ sex * season + sex * RIB	324.63	MV ~ sex + RIB * MB	199.16
MV ~ sex + season * RIB	326.51	MV ~ sex * MB + RIB	199.86
MV ~ sex * RIB + season	326.46	MV ~ sex * MB + sex * RIB	192.68
MV ~ sex * season * RIB	324.67	MV ~ sex * MB * RIB	186.24
**MCP Models**			
MCP ~ sex + season + RIB	283.64	MCP ~ sex + MB + RIB	178.18
MCP ~ sex * season + RIB	286.22	MCP ~ sex * RIB + MB	173.97
MCP ~ sex * season + sex * RIB	284.92	MCP ~ sex + RIB * MB	179.60
MCP ~ sex + season * RIB	285.49	MCP ~ sex * MB + RIB	179.67
MCP ~ sex * RIB + season	282.46	MCP ~ sex * MB + sex * RIB	171.13
MCP ~ sex * season * RIB	286.75	MCP ~ sex * MB * RIB	166.46

**TABLE A2:** Akaike Information Criterion (AIC) model fit scores for accelerometer activity intensity metric models including sex (male, female), season (mating, non‐mating), body temperature (BT), snout‐vent length (SVL), and road interaction category (RIB) as fixed effects. Models included the overall dynamic body acceleration (ODBA) and the vector of the dynamic body acceleration (VeDBA) as response variables.

Accelerometer Activity Metric Models	
Overall Dynamic Body Acceleration Models	AIC
ODBA ~ sex + RIB + SVL + BT + season	1816.83
ODBA ~ sex * RIB + SVL + BT + season	1814.48
ODBA ~ sex + RIB * season + SVL + BT	1817.75
ODBA ~ sex * RIB * season + SVL + BT	1821.19
**Vector of Dynamic Body of Acceleration Models**	**AIC**
VeDBA ~ sex + RIB + SVL + BT + season	1788.84
VeDBA ~ sex * RIB + SVL + BT + season	1786.49
VeDBA ~ sex + RIB * season + SVL + BT	1789.75
VeDBA ~ sex * RIB * season + SVL + BT	1793.29

**TABLE A3:** Coefficients, standard error, and *p*‐values for post hoc estimated marginal means pairwise comparisons of RT‐derived road interaction (RIB) and behavioral season (Non‐mating, Mating) model parameters. Coefficients can be used to interpret the direction of factor levels.

Interaction contrast	Reference level	Estimate	SE	df	*t* ratio	*p*
**MPD**						
Female—Male	Mating	−0.69	0.146	60.6	−4.69	< 0.0001*
	Non‐mating	−0.24	0.173	77.7	−1.4	0.29
Mating—Non‐mating	Female	0.002	0.126	65.8	0.015	0.99
	Male	0.44	0.125	67.2	3.56	0.001*
**DPM**						
Female—Male	Mating	−0.606	0.118	60	−5.11	< 0.0001*
	Non‐mating	−0.287	0.14	78	−2.05	0.08
Mating—Non‐mating	Female	−0.257	0.167	47.3	−1.538	0.24
	Male	−0.195	0.196	50.3	−0.997	0.54
**MV**						
Female*–*Male	Mating	−1.42	0.305	66.5	−4.64	< 0.0001*
	Non‐mating	−0.57	0.375	82.4	−1.52	0.24
Mating – Non‐mating	Female	−0.207	0.306	67	−0.68	0.75
	Male	0.639	0.297	69.5	2.15	0.07
**MCP**						
Female *–* Male	Mating	−0.88	0.306	53.1	−2.88	0.01*
	Non‐mating	−0.909	0.346	68.8	−2.625	0.02*
Mating – Non‐mating	Female	−0.124	0.21	62.4	−0.59	0.80
	Male	−0.152	0.21	63.3	−0.73	0.72

* denotes statistical significance for factor level pairwise contrasts.

**TABLE A4:** MPD. Coefficients, standard error, and P‐values for post hoc estimated marginal means pairwise comparisons of RT‐derived road interaction (RIB) and mate location success (MB) model parameters. Reference levels for main effect parameters are in parentheses (Male, MB 0 [No], RIB 0 [No]), and coefficients can be used to interpret the direction of the factor level.

Interaction contrast	Reference level	Estimate	SE	df	*t* ratio	*p*
**Sex** * **MB**						
Female – Male	MB 0	−0.61	0.158	43.8	−3.867	0.0007*
	MB 1	−0.84	0.213	50.5	−3.941	0.0005*
MB 0–MB 1	Male	−0.408	0.229	49.9	−1.78	0.0005*
	Female	−0.636	0.16	54.4	−3.985	0.15
**Sex** * **RIB**						
Female – Male	RIB 0	−0.693	0.14	38	−4.935	< 0.0001*
	RIB 1	−0.755	0.217	47.9	−3.483	0.002*
RIB 0 – RIB 1	male	−0.257	0.167	47.3	−1.538	0.24
	female	−0.195	0.196	50.3	−0.997	0.54
**MB** * **RIB**						
MB 0–MB 1	RIB 0	−0.318	0.149	52.8	−2.138	0.07
	RIB 1	−0.725	0.226	52.7	−3.213	0.005*
RIB 0 – RIB 1	MC 0	−0.0232	0.152	50.9	−0.153	0.99
	MC 1	−0.4297	0.205	49.7	−2.098	0.08
**Sex** * **MB** * **RIB**						
MB 0–MB 1 (male)	RIB 0	−0.7161	0.209	49.8	−3.433	0.005*
	RIB 1	−0.555	0.244	43.8	−2.271	0.11
MB 0–MB 1 (female)	RIB 0	0.079	0.2	47.4	0.396	0.99
	RIB 1	−0.895	0.376	52.1	−2.383	0.08

* denotes statistical significance for factor level pairwise contrasts.

**TABLE A5:** DPM. Coefficients, standard error, and P‐values for post hoc estimated marginal means pairwise comparisons of RT‐derived road interaction (RIB) and mate location success (MB) model parameters. Reference levels for main effect parameters are in parentheses (Male, MB 0 [No], RIB 0 [No]), and coefficients can be used to interpret the direction of factor levels.

Interaction contrast	Reference level	Estimate	SE	df	*t* ratio	*p*
**Sex** * **MB**						
Female–Male	MB 0	−0.538	0.142	42.1	−3.79	0.0009*
	MB 1	−0.733	0.194	50.3	−3.78	0.0008*
MB 0–MB 1	Male	−0.524	0.149	47.9	−3.52	0.002*
	Female	−0.329	0.209	51	−1.57	0.23
**Sex** * **RIB**						
Female–Male	RIB 0	−0.604	0.125	36	−4.83	0.0001*
	RIB 1	−0.67	0.197	48.1	−3.39	0.003*
RIB 0–RIB 1	Male	−0.26	0.151	43.9	−1.72	0.18
	Female	−0.197	0.179	50.1	−1.101	0.47
**MB** * **RIB**						
MB 0–MB 1	RIB 0	−0.27	0.136	51.2	−1.96	0.11
	RIB 1	−0.587	0.21	52.1	−2.81	0.01*
RIB 0–RIB 1	MC 0	−0.07	0.139	50.9	−0.483	0.86
	MC 1	−0.39	0.187	49.4	−2.08	0.08
**Sex** *MB* **RIB**						
MB 0–MB 1 (male)	RIB 0	−0.63	0.192	50.6	−3.25	0.008*
	RIB 1	−0.42	0.23	46.5	−1.85	0.25
MB 0–MB 1 (female)	RIB 0	0.09	0.19	45.7	0.515	0.97
	RIB 1	−0.75	0.344	51.9	−2.18	0.13

* denotes statistical significance for factor level pairwise contrasts.

**TABLE A6:** MCP. Coefficients, standard error, and *p*‐values for post hoc estimated marginal means pairwise comparisons of RT‐derived road interaction (RIB) and mate location success (MB) model parameters. Reference levels for main effect parameters are in parentheses (Male, MB 0 [No], RIB 0 [No]), and coefficients can be used to interpret the direction of factor levels.

Interaction contrast	Reference level	Estimate	SE	df	*t* ratio	*p*
**Sex** * **MB**						
Female–Male	MB 0	−0.501	0.212	41.6	−2.36	0.04*
	MB 1	−0.47	0.276	49.9	−1.69	0.18
MB 0–MB 1	Male	−0.37	0.218	49.7	−1.69	0.18
	Female	−0.402	0.303	43.6	−1.33	0.34
**Sex** * **RIB**						
Female–Male	RIB 0	−0.304	0.173	30.8	−1.76	0.17
	RIB 1	−0.67	0.288	47.7	−2.31	0.05*
RIB 0–RIB 1	Male	−0.33	0.208	36	−1.59	0.22
	Female	−0.03	0.266	49.4	0.111	0.99
**MB** * **RIB**						
MB 0–MB 1	RIB 0	−0.29	0.19	46.4	−1.52	0.25
	RIB 1	−0.48	0.308	50.7	−1.56	0.23
RIB 0–RIB 1	MC 0	−0.06	0.213	48.5	−0.264	0.96
	MC 1	−0.25	0.264	48.6	−0.93	0.59
**Sex** * **MB** * **RIB**						
MB 0–MB 1 (male)	RIB 0	−1.611	0.425	50.3	−3.79	0.002*
	RIB 1	−0.377	0.501	44.9	−0.75	0.91
MB 0–MB 1 (female)	RIB 0	0.26	0.404	47	0.637	0.95
	RIB 1	−1.11	0.76	52.2	−1.45	0.48

* denotes statistical significance for factor level pairwise contrasts.

**TABLE A7:** MV. Coefficients, standard error, and *p*‐values for post hoc estimated marginal means pairwise comparisons of RT‐derived road interaction (RIB) and mate location success (MB) model parameters. Reference levels for main effect parameters are in parentheses (Male, MB 0, RIB 0), and coefficients can be used to interpret the direction of factor levels.

Interaction contrast	Reference level	Estimate	SE	df	*t* ratio	*p*
**Sex** * **MB**						
Female–Male	MB 0	−1.18	0.369	41.5	−3.199	0.005*
	MB 1	−2.02	0.511	50.8	−3.949	0.0005*
MB 0–MB 1	Male	−1.568	0.397	49.7	−3.95	0.0005*
	Female	−0.733	0.55	51	−1.334	0.34
**Sex** * **RIB**						
Female–Male	RIB 0	−1.41	0.324	34.7	−4.36	0.0002*
	RIB 1	−1.79	0.516	48.6	−3.46	0.002*
RIB 0–RIB 1	Male	−0.66	0.392	42	−1.69	0.19
	Female	−0.29	0.472	50.5	−0.611	0.79
**MB** * **RIB**						
MB 0–MB 1	RIB 0	−0.82	0.36	50.3	−2.298	0.05*
	RIB 1	−1.5	0.55	52.4	−2.67	0.02*
RIB 0–RIB 1	MC 0	−0.15	0.37	50.9	−0.396	0.91
	MC 1	−0.806	0.491	49.6	−1.643	0.20
**Sex** * **MB** * **RIB**						
MB 0–MB 1 (male)	RIB 0	−2.06	0.51	50.8	−4.05	0.0007*
	RIB 1	−1.08	0.61	48.5	−1.8	0.29
MB 0–MB 1 (female)	RIB 0	0.42	0.47	44.2	0.89	0.84
	RIB 1	−1.89	0.91	52.4	−2.08	0.16

* denotes statistical significance for factor level pairwise contrasts.
